# Effect of Ascorbic Acid on Noise Induced Hearing Loss in Rats 

**Published:** 2015-07

**Authors:** Ziba Loukzadeh, Abolfazl Hakimi, Mansour Esmailidehaj, Amir Houshang Mehrparvar

**Affiliations:** 1 *Industrial Diseases Research Center, Shahid Sadoughi University of Medical Sciences, Yazd, Iran.*; 2 *Department of Physiology, Faculty of Medicine, Shahid Sadoughi University of Medical Sciences, Yazd, Iran.*

**Keywords:** Ascorbic acid, Hearing loss, Noise, Otoacoustic emission.

## Abstract

**Introduction:**

After presbycusis, noise-induced hearing loss is the second most common cause of acquired hearing loss. Numerous studies have shown that high-intensity noise exposure increases free radical species; therefore, use of antioxidants to detoxify the free radicals can prevent cellular damage in the cochlea. We studied the potential hearing protective effect of different doses of ascorbic acid administered prior to noise exposure in rats.

**Materials and Methods:**

Twenty-four male albino Wistar rats were randomly allocated into four groups: groups A, B, and C received 1250, 250, and 50 mg/kg/day of ascorbic acid, respectively, and group D acted as the control group. After 14 days of ascorbic acid administration, the rats were exposed to noise (105 dB sound pressure level for 2 h). Distortion product otoacoustic emissions (DPOAE) were recorded prior to starting the ascorbic acid as baseline and 1 h after the noise exposure.

**Results:**

The amplitude decrease was 14.99 dB for group A, 16.11 dB for group B, 28.82 dB for group C, and 29.91 dB for the control group. Moderate and high doses of ascorbic acid significantly reduced the transient threshold shift in the rats.

**Conclusion:**

The results of present study support the concept of cochlea protection by antioxidant agents. This dose-dependent protective effect was shown through the use of ascorbic acid treatment prior to noise exposure.

## Introduction

The most common hazardous exposure at workplace is noise exposure (1). It is estimated that more than 9 million Americans are exposed to occupational noise levels above 85 dB (2). Approximately 16% of disabling hearing loss is caused by excessive noise exposure in the workplace and after presbycusis (3). Noise-induced hearing loss (NIHL) is second most common cause of acquired hearing loss (4). Given that there is no known treatment for this irreversible condition, prevention of NIHL is the current management goal. 

The precise mechanism which leads to NIHL is unknown. Numerous studies have shown that high-intensity noise exposure causes mechanical or metabolic changes in the cochlea, leading to inner-ear damage (5). The metabolic changes are caused mainly by an increase in free radical species (FRS) that play a primary role in NIHL (6). Harmful effects of FRS are generally prevented by endogenous antioxidants; but since endogenous antioxidant capacity is limited, the inactivation of FRS by antioxidant administration has recently been studied widely (7).

Many studies have been conducted in experimental animals with different noise exposure duration and intensity using measuring methods such as auditory brainstem response (ABR) and otoacoustic emission (OAE) (6-13). Different antioxidants have also been examined, such as N-acetyl-cysteine, vitamin A, magnesium, carnitine, administered either before or after noise exposure (5-15). Ascorbic acid (AA) is a water-soluble antioxidant with broad-spectrum activity (16), use of which seems to prevent low-density lipoprotein oxidation (5). NIHL is associated with DNA oxidation and lipid peroxidation in the cochlea, and AA may reduce NIHL by inhibition of these types of oxidative damage (12). A study by Derekoy et al. showed that administration of AA in the rabbit 2.5 days before noise exposure was effective in preventing transient threshold shift (TTS) (5). Moreover, treatment with AA 7 and 35 days before noise exposure in the guinea pig decreased susceptibility to NIHL (7,13).

To the best of our knowledge, no study has evaluated the effect of AA on prevention of NIHL in rats. Therefore, we studied the potential hearing protective effect of different doses of AA administered prior to noise exposure in rats. 

## Materials and Methods

*Animals*

This clinical trial was conducted in 24 male albino Wistar rats aged 2–3 months and weighing 250–300 g at the beginning of the study. Animals were housed in polycarbonyl cages (15 × 20 × 30 cm) in a room in which ambient noise levels were less than 45 dB with a 12-h light/dark cycle and a room temperature and humidity of 25 °C and 55%, respectively. Animals had free access to water and food during the day, and the care and handling of the animals were in accordance with National Institutes of Health (NIH) guidelines. This study was approved by the Ethics Committee of Shahid Sadoughi University of Medical Sciences (Ethics Committee approval number: 112611; date: April/27/2012).

*Experimental groups*

Normal hearing rats were randomly allocated into four groups (n=6 for each group). Before the administration of AA for measurement of baseline distortion product otoacoustic emissions (DPOAE), rats with absent DPOAE amplitude or rats with DPOAE that were not consistent with laboratory norms were excluded. Groups A, B, and C received 1250, 250, and 50 mg/kg/day of AA (Osvah Pharmaceutical Co., Iran), respectively. A 1-ml volume solution was prepared by diluting the different doses of AA in normal saline and administering by gavage once a day for 14 days before noise exposure. The control group also received 1 ml normal saline/day by gavage for 14 days. 

*Noise exposure*

After 14 days of drug administration, animals were exposed to 3 kHz octave band noise at a sound pressure level of 105 dB for 2 h. Noise exposure was conducted in a glass chamber (1 × 1 × 1 m) containing six polycarbonyl cages (15 × 20 × 20 cm). The noise was generated from a Hi-Fi amplifier (Model AP12 PEJVAK AVA Co., Iran) using two speakers on the sides of the glass chamber. Sound intensity was measured by a calibrated sound level meter (TES-1358 Sound Analyze, Taiwan) placed at the level of the external auditory canal of the rats.

*Auditory assessment*

The hearing level of each rat was evaluated by measuring DPOAE amplitudes (Madsen Orbiter 922 Version 2, Denmark). Each rat was tested by DPOAE in two steps: prior to starting the AA as baseline and 1 h after the noise exposure to measure TTS (6). The DPOAE amplitudes were tested at frequencies of 2, 3, 4, 6, and 8 kHz. Before each recording session, rats were anesthetized with an intraperitoneal injection of 10 mg/kg xylazine (Alfasan Diergenee- smiddelen BV, Woerden)and 40 mg/kg ketamine(Alfasan Diergenee-smiddelen BV, Woerden) using an insulin syringe. To maintain body temperature at ~37°C during recordings, animals were placed on a heating pad. 

*Statistical analysis*

All data were analyzed using GraphPad Prism 5 (San Diego, CA) and presented as mean ± SEM. To compare the mean difference among groups, a repeated measure two-way analysis of variance (ANOVA) was used. P<0.05 was considered statistically significant. 

## Results

**Fig1:**
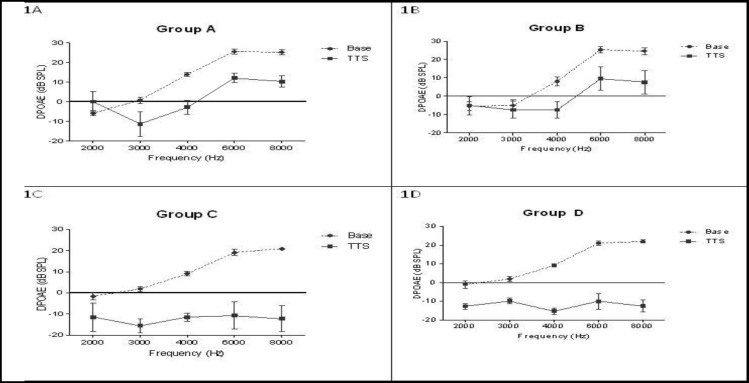
Comparison of distortion product otoacoustic emissions (DPOAE) amplitudes (mean ± SEM) measured at the start of the experiment (baseline amplitudes) (dotted lines) and 1 h after noise exposure (transient threshold shift: TTS) (solid lines) in four study groups (1A: group A, 1B: group B, 1C: group C, 1D: group D or control). Groups A, B, and C received 1250, 250, and 50 mg/kg/day of ascorbic acid, respectively. Transient threshold shift at 4–8 KHz was significantly lower for groups A and B

Mean DPOAE amplitudes ranged from −5.8 to 25.5 dB across all frequencies and there were no differences in baseline DPOAE at any frequency among the groups. After the noise exposure, there was a significant decrease in hearing levels at frequencies of 4, 6, and 8 kHz in all groups compared with the same baseline hearing level (P < 0.05). The amplitude decrease was 14.99 dB for group A, 16.11 dB for group B, 28.82 dB for group C, and 29.91 dB for the control group (group D) (Fig. 1).

**Fig 2:**
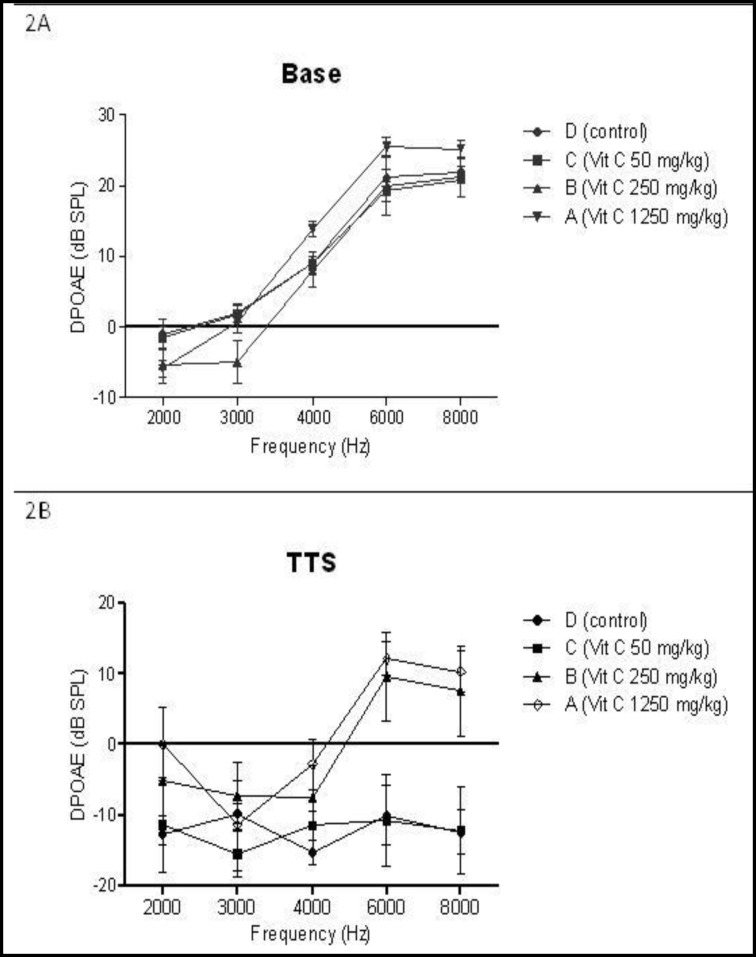
Baseline amplitude (2A) and changes in amplitudes 1 h after noise exposure (transient threshold shift: TTS) (2B) measured using distortion product otoacoustic emissions (DPOAE). All results are presented as mean ± SEM. There was no significant amplitude difference among the groups before noise exposure. After noise exposure, mean amplitudes at 4–8 KHz were significantly higher for groups A and B compared with the control group

1 h after the noise exposure, the decrease in DPOAE amplitude after exposure to noise in group C (50 mg/kg of AA) was similar to that in the control group at all frequencies (P>0.05). However, the amplitude shifts at frequencies of 6 and 8 kHz in the groups receiving higher doses of AA (groups A and B) were significantly lower than those in the control group (P<0.01) (Fig.2). 

Mean post-exposure DPOAE amplitudes at 4–8 KHz in groups A and B were 19.08 and 15.78 dB, respectively; significantly higher than those in the control group (P<0.05). The threshold shift after the noise exposure in group A was less than that in group B, but this difference was not statistically significant (P>0.05).

No apparent side effects of AA were seen in the rats over the course of the experiment.

## Discussion

We evaluated the hearing protective effect of pre-exposure administration of AA at different dosages (50, 250, and 1250 mg/kg/day) in rats. The results suggest that moderate and high doses of AA lead to a significant decrease in TTS among the rats. A previous survey performed on guinea pigs showed that pre-exposure use of AA resulted in minimal or no loss of hair cells within the cochlea (17). Derekoy et al. suggested that a brief application of AA (500 mg twice daily for 2.5 days) before noise exposure can decrease TTS in rabbits, as well as resulting in a significant decrease in the formation of FRS (5). In other studies, pre-exposure administration of AA in the guinea pig dose-dependently reduced permanent threshold shift (PTS) (7,13). However, Rabinowitz et al. examined the role of a number of metabolic factors such as AA on human cochlear function in 58 noise-exposed workers, and found no protective effect for AA (18).

It has been suggested that as well as being dose-dependent, the protective effects of antioxidant drugs against NIHL are also time-dependent. In this context, Lorito et al. recommended that antioxidant treatment should take place 7 days or even 14 days prior to noise exposure (6). Kopke et al. suggested that the existence of antioxidants before exposure to noise can cause shifts in the lower hearing threshold as well as hair-cell death (19). In our study, as also shown in other studies (5,7,13), administration of AA before noise exposure had a protective effect against NIHL.

Furthermore, as in previous studies (7,13), it was shown in the present study that higher doses of AA (250 and 1250 mg/kg/day) could reduce threshold shift compared with the control group. Although threshold shift after noise exposure in the highest dose of AA (1250 mg/kg/day) was less than that in the moderate dose (250 mg/kg/day), this difference was not statistically significant.

Some mechanisms for AA hearing protection have been proposed. Tripeptide glutathione (GSH) as a major intracellular antioxidant is proven to detoxify free radicals and attenuate reactive oxygen species (ROS) and/or reactive nitrogen species and to prevent NIHL (20). A study has shown that there are significant interrelationships between GSH and AA, and that AA can probably protect NIHL in cooperation with GSH (13). AA treatment could inhibit both lipid peroxidation and protein oxidation in rabbits exposed to noise (5). 

We could not confirm the protective effect of AA in the high-frequency range (10–40 KHz) and other noise exposure paradigms.

AA is clinically utilized and orally administered in humans. Although the results of the present study may be applicable to humans, further studies are needed to examine the effect of AA in other experimental and/or clinical settings in order to better elucidate the optimal dose and time for administration of AA. 

## Conclusion

The results of the present study support the concept of cochlea protection by use of antioxidant agents. This protective effect was shown in AA treatment before exposure to noise and was dose-dependent.
